# Catechol-Functional Chitosan/Silver Nanoparticle Composite as a Highly Effective Antibacterial Agent with Species-Specific Mechanisms

**DOI:** 10.1038/s41598-017-02008-4

**Published:** 2017-05-12

**Authors:** Xiaofei Huang, Xiaojiong Bao, Yalan Liu, Zhengke Wang, Qiaoling Hu

**Affiliations:** 1 MOE Key Laboratory of Macromolecular Synthesis and Functionalization, Department of Polymer Science and Engineering, Zhejiang University, Hangzhou, 310027 China; 2 Key Laboratory of Adsorption and Separation Materials & Technologies of Zhejiang Province, Hangzhou, 310027 China

## Abstract

In this study, silver nanoparticles (Ag NPs) coated with catechol-conjugated chitosan (CSS) were prepared using green methods. Interestingly, we uncovered that CSS-coated Ag NPs (CSS-Ag NPs) exhibited a higher toxicity against gram-negative *Escherichia coli* (*E. coli*) bacteria than against gram-positive Staphylococcus aureus (S. aureus) bacteria. The differences revealed that the CSS-Ag NPs killed gram bacteria with distinct, species-specific mechanisms. The aim of this study is to further investigate these underlying mechanisms through a series of analyses. The ultrastructure and morphology of the bacteria before and after treatment with CSS-Ag NPs were observed. The results demonstrated the CSS-Ag NPs killed gram-positive bacteria through a disorganization of the cell wall and leakage of cytoplasmic content. In contrast, the primary mechanism of action on gram-negative bacteria was a change in membrane permeability, induced by adsorption of CSS-Ag NPs. The species-specific mechanisms are caused by structural differences in the cell walls of gram bacteria. Gram-positive bacteria are protected from CSS-Ag NPs by a thicker cell wall, while gram-negatives are more easily killed due to an interaction between a special outer membrane and the nanoparticles. Our study offers an in-depth understanding of the antibacterial behaviors of CSS-Ag NPs and provides insights into ultimately optimizing the design of Ag NPs for treatment of bacterial infections.

## Introduction

Nowadays, antibacterial agents are widely used in treating infectious diseases caused by pathogenic bacteria. However, the wide use of antibiotics has led to a rise in microbial drug resistance, resulting in poor treatment efficacy and significant economic loss^1–3^. There is an urgent need to develop new antibacterial agents in place of antibiotics.

In the past few years, silver nanoparticles (Ag NPs) have been considered potential antibacterial agents owing to their broad-spectrum antimicrobial activity^4, 5^. It has been confirmed that the antibacterial properties of Ag NPs depend on several physicochemical properties of the particles, including their size, shape, chemistry, and surface coating^6, 7^. Among these physicochemical properties, the surface coating plays a critical role in biocompatibility and the toxicity of silver nanoparticles^8^. In our previous reported work, Ag NPs coated with a chitosan derivative (catechol-conjugated chitosan, CSS) were prepared using green methods^9^. The CSS-coated Ag NPs (CSS-Ag NPs) exhibited notable antibacterial activities on the bacteria due to their uniform size and excellent stability. In addition, the biocompatible polymer coating of the Ag NPs led to low cytotoxicity on human cells. Thus, these CSS-Ag NPs with effective antibacterial activity and low cytotoxicity represented a potential candidate for use in biological and pharmaceutical fields to prevent infections caused by microorganisms. However, a problem remains, which is that the antibacterial mechanisms of CSS-Ag NPs toward different types of bacteria needs to be figured out urgently in order to use these antibacterial agents in practice.

Realistically speaking, this is a problem not only for CSS-Ag NPs, but also for all kinds of Ag NPs. The dramatic expansion in the applications for Ag NPs means it is necessary to figure out the antibacterial mechanisms involved. Despite numerous reports, there is no consensus regarding the antibacterial mechanisms of Ag NPs^10^. Recently, some researchers have tried to use electron microscopy to observe the antibacterial behaviors of Ag NPs^11–13^. The changes in the cellular ultrastructure of the bacteria provide useful insight into the action mechanism of Ag NPs. These results inspired us to further investigate the antibacterial mechanism of CSS-Ag NPs using electron microscopy. This approach will enable us to apply efficient and safe CSS-Ag NPs to the treatment of bacterial infections with an in-depth understanding of the mechanisms.

**Figure 1:**
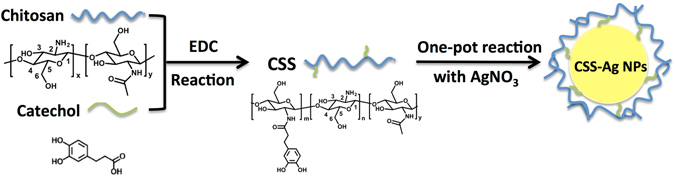
Schematic representation for the green synthesis of CSS-Ag NPs. CSS was synthesized by the conjugation of bio-inspired catechol to chitosan through EDC chemistry procedure. As a stabilizing and reducing agent, CSS was added into Ag NO_3_ solution to prepare CSS-Ag NPs.

In this study, we synthesized CSS-Ag NPs according to our prior work. A schematic of the green synthesis process is shown in Fig. 1. The antibacterial activities toward Escherichia coli (*E. coli*) and Staphylococcus aureus (S. aureus) were evaluated. Interestingly, we uncovered that the CSS-Ag NPs exhibit a higher toxicity against gram-negative (G−) *E. coli* bacteria than against gram-positive (G+) S. aureus bacteria. The differences revealed the species-specific mechanisms of CSS-Ag NPs toward G+ and G− bacteria. We utilized transmission electron microscopes (TEMs) and scanning electron microscopes (SEMs) to observe the bacteria before and after exposure to CSS-Ag NPs, respectively. A BacLight LIVE/DEAD membrane permeability kit was used to evaluate the membrane permeability of the bacteria. In addition, the leakage of proteins through the bacteria membrane was detected by a Bicinchoninic acid (BCA) protein assay kit.

## Results and Discussion

### Synthesis and characterization of CSS and CSS-Ag NPs

**Figure 2:**
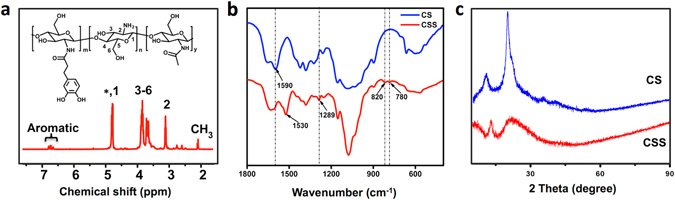
Characterization of CSS. (**a**) ^1^H NMR spectrum of CSS, (**b**) FT-IR spectrum of CS and CSS, (**c**) XRD analysis of CS and CSS.

Based on our previous work, CSS, a highly soluble chitosan derivative, was synthesized by conjugation of catechol to chitosan, which was confirmed by NMR, FT-IR, and XRD^9^. As shown in Fig. 2a, the aromatic protons featured in catechol were successfully detected in the ^1^H NMR spectrum of CSS. Fig. 2b shows the FT-IR spectrum for CS and CCS. The CSS spectrum shows several new absorption peaks at 1289, 820, and 780 cm^−1^, corresponding to the characteristic absorption peaks of phenolic structures^14^. The characteristic absorption peak (–NH_2_ group) at 1590 cm^−1^ decreased dramatically and the absorption peak (amide type II) at 1530 cm^−1^ increased in intensity, revealing that the amino groups on chitosan reacted with the catechol to form amide^15^. XRD analysis was carried out to further investigate the phase structural changes from CS to CCS. As shown in Fig. 2c, XRD pattern analysis of CS shows two peaks at 12° and 21° corresponding to the hydrated crystals and anhydrous crystals, respectively. Therein, the remarkable peak at 21° is attributed to strong hydrogen bonds within or between the molecules of CS^16^. As shown in the XRD pattern analysis of CSS, the peak at 21° was weakened dramatically, revealing a partial breakage of the hydrogen bonds. The results indicated that catechol conjugation would significantly decrease the rigidity of chitosan backbones, which contribute to the significantly enhanced water-solubility of CSS. As a reducing and stabilizing agent, CSS was added to a silver nitrate aqueous solution to prepare the CSS-Ag NPs. A schematic of the green synthesis process is shown in Fig. 1.

**Figure 3:**
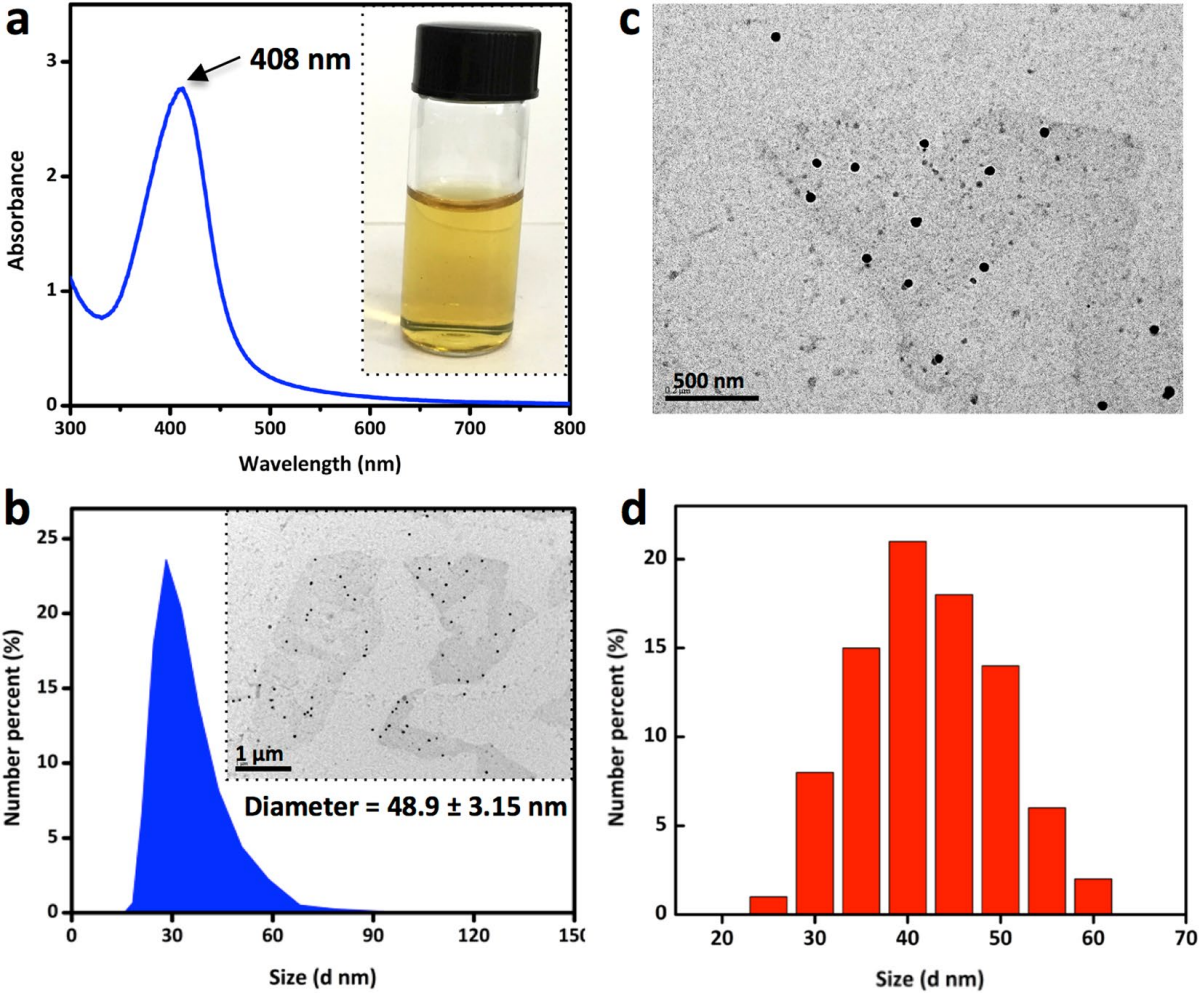
Dispersion property of CSS-Ag NPs solution. (**a**) UV-vis spectrum and optical photo of CSS-Ag NPs solution, (**b**) size distribution of CSS-Ag NPs measured by DLS, and insert shows a TEM image of CSS-Ag NPs, (**c**) TEM image of CSS-Ag NPs, (**d**) size distribution of CSS-Ag NPs measured by TEM.

Figure 3a shows the UV-vis spectrum and corresponding optical photo of the synthesized CSS-Ag NP solution. A characteristic silver surface plasmon resonance (SPR) absorption band was observed at 408 nm, indicating a successful formation of silver nanoparticles without aggregation^8^. The transparent yellow of the CSS-Ag NP solution also confirmed the UV-vis result.

Figure 3b–d shows the size distribution of CSS-Ag NPs measured by TEM and DLS. The TEM image indicates that the CSS-Ag NPs were spherical with a narrow size distribution, which coincided with the DLS result. The hydrodynamic diameter of the CSS-Ag NPs evaluated by DLS was 48.9 ± 3.15 nm. The average size of the CSS-Ag NPs obtained from the TEM images was 44.2 ± 5.2 nm.

### Antibacterial activities of CSS-Ag NPs

**Figure 4:**
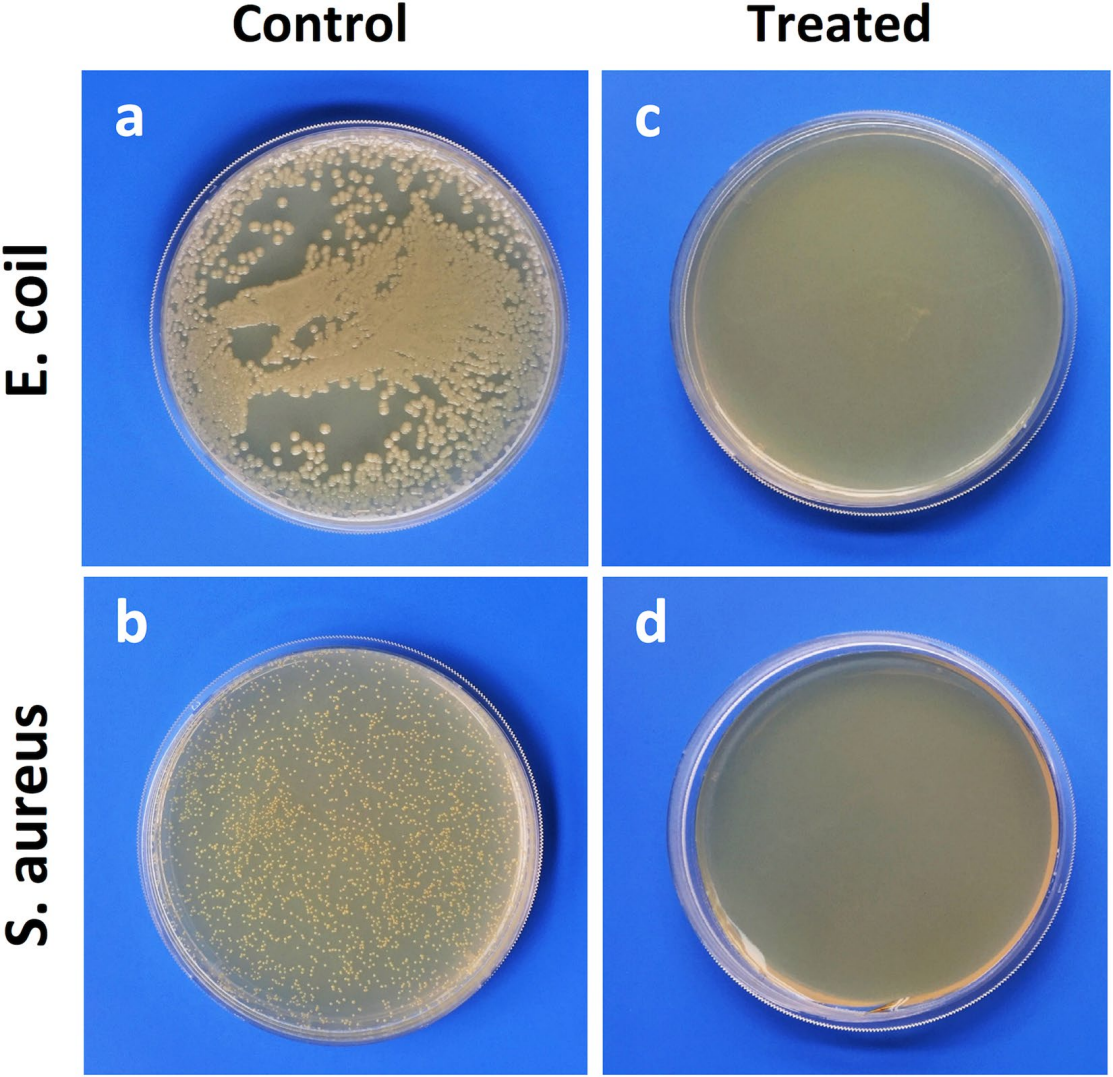
Images of CFU counting. (**a,b**) Untreated E. coil and S. aureus; (**c**) E. coil treated with CSS-Ag NPs (14 μg/ml, silver concentration); (**d**) S. aureus treated with CSS-Ag NPs (25 μg/ml, silver concentration).

In our prior study, the minimum inhibitory concentration (MIC) of CSS-Ag NPs was measured to characterize the antibacterial activity, which was defined as the lowest concentration sufficient to prevent bacterial growth^17^. If we want to further investigate how CSS-Ag NPs can kill bacteria, we need to coculture the bacteria with a minimum bactericidal concentration of nanoparticles to make sure all cells are killed. Therefore, the minimum bactericidal concentrations (MBCs) of CSS-Ag NPs were measured by counting colony-forming units (CFUs). As shown in Fig. 4, no viable colony remained on the agar plate after exposure to CSS-Ag NPs at low concentration, indicating that the cells were killed. This demonstrated that CSS-Ag NPs exhibited remarkable antibacterial activities at very low dosages, with an MBC of 14 μg/mL against *E. coli* and 25 μg/mL against S. aureus.

### Ultrastructural changes of the bacteria after exposure to CSS-Ag NPs

Interestingly, the MBC values revealed that the CSS-Ag NPs exhibited a higher toxicity against G− than against G+ bacteria. We were very curious about the precise antibacterial mechanism that might explain the efficient and species-specific antibacterial activities of CSS-Ag NPs. However, the mechanism has not been deciphered although it is crucial for the application of CSS-Ag NPs. Some investigations in the literature have reported observation of changes in the cellular ultrastructure that could help decipher the antibacterial mechanism exerted by nanoparticles^11^. Therefore, we utilized TEM to further investigate the changes in the bacteria before and after exposure to CSS-Ag NPs.

**Figure 5:**
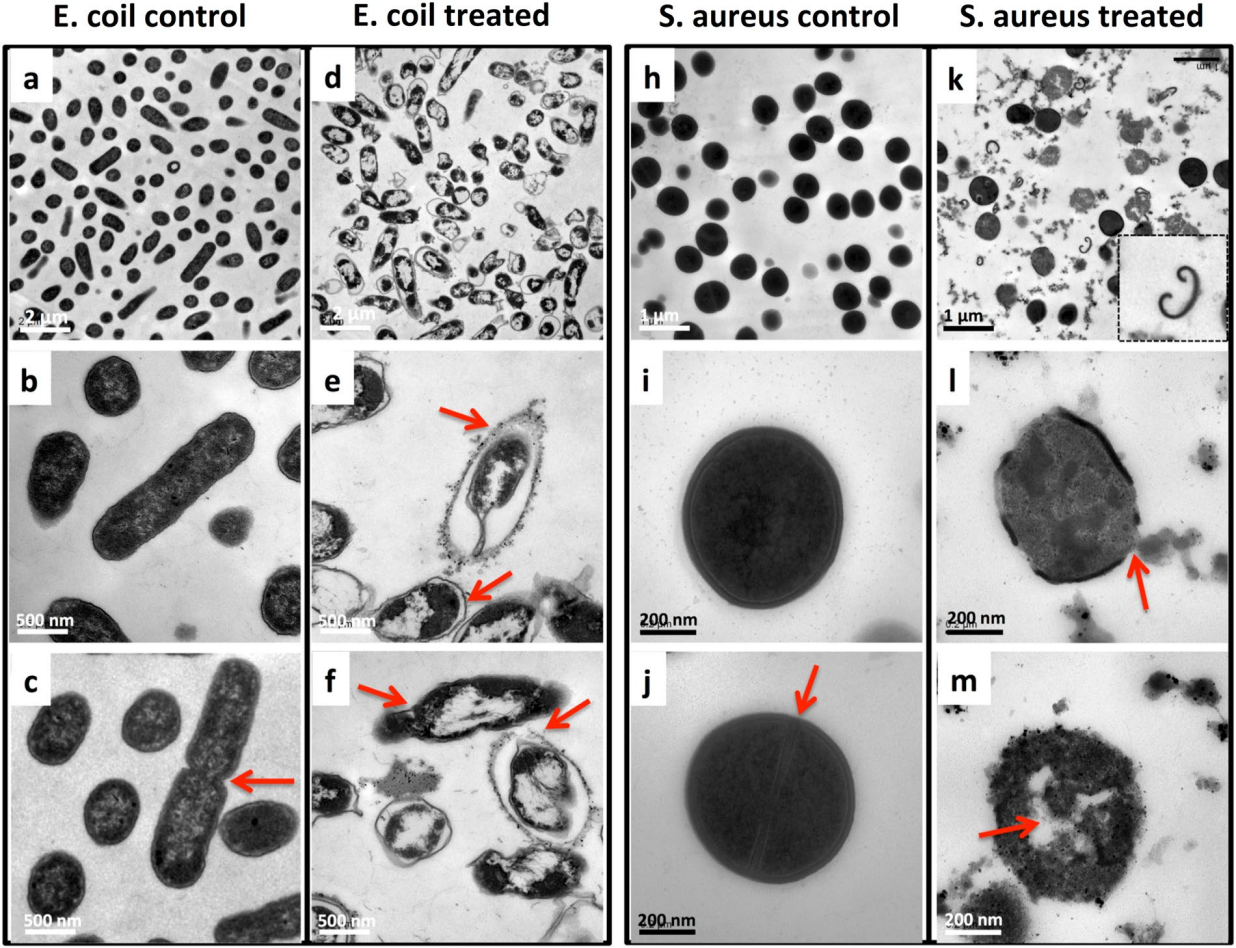
TEM images of bacteria treated with or without CSS-Ag NPs for 8 h. (**a–c**) Untreated E. coil; (**d–f**) treated E. coil; (**h–j**) untreated S. aureus; (**k–m**) treated S. aureus.

**Figure 6:**
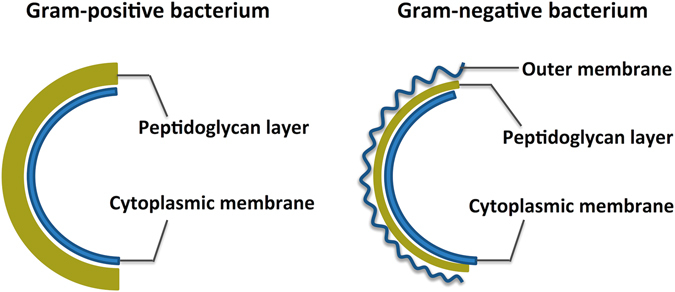
Structure schematic of Gram-positive and Gram-negative bacterium.

The untreated bacterial cells (*E. coli* and S. aureus) showed an intact cell membrane and wall (Fig. 5a–c,h–j **)**. It was clear that the cells had a uniform electron density, suggesting that the cells were in a normal condition. Cell division was observed (Fig. 5c,j), which means the cellular proliferation of bacteria remained normal. By comparing Fig. 5b,i, we found a significant difference in the cell wall between *E. coli* and S. aureus. In fact, the bacteria are simply divided into G+ and G− based on the structural difference in cell walls^18^. As shown in Fig. 6, G− bacteria have a thin peptidoglycan layer (about 2–3 nm) between the cytoplasmic membrane and the outer membrane. In contrast, G+ bacteria lack this outer membrane, but have a thicker peptidoglycan layer (about 30 nm).

Figure 5d–f shows the ultrastructure of *E. coli* to be remarkably changed after exposure to CSS-Ag NPs. Many nanoparticles attached to the surface of the *E. coli*, and a serious detachment of the cell wall from the outer membrane was observed (Fig. 5e, red arrow). A low-density region was found in the center of almost all the cells, suggesting the cytoplasm was seriously damaged. As a first line of defense, the outer membrane of the *E. coli* was then disintegrated by the nanoparticles (Fig. 5f, red arrow). These cells without an outer membrane still maintained the cytoplasmic shape and no leakage of cytoplasmic contents could be observed (Fig. 5f, red arrow).

The ultrastructure of S. aureus treated with CSS-Ag NPs was also changed considerably. Figure 5k shows some cell walls that fell off from the cells. Figure 5l revealed a disorganization of the bacterial cell wall and leakage of cytoplasmic contents (red arrow). Those bacterial cells without a wall and membrane swelled and nanoparticles could be observed inside the cytoplasm (Fig. 5m, red arrow). However, a fraction of the cells that maintained a complete cell wall still had a uniform electron density (Fig. 5k).

There was no cell proliferation of *E. coli* and S. aureus observed after exposure to CSS-Ag NPs (Fig. 5d,k **)**. This result demonstrated that CSS-Ag NPs could inhibit the proliferation of two kinds of bacteria, which may be part of the reason for the remarkable antibacterial activities.

The significant difference in cellular ultrastructure revealed that CSS-Ag NPs killed the two kinds of bacteria through different pathways. The species-specific antibacterial mechanism may be caused by structural differences in the organization of the bacterial cell wall. Some studies found that the thicker cell wall of G+ bacteria acts as a barrier protecting the cell from penetration of silver nanoparticles and ions into the cytoplasm^19, 20^. This may be part of the reason CSS-Ag NPs exhibited a better antibacterial effect on G− bacteria rather than G+ bacteria. The speculation can be confirmed by comparing the ultrastructural changes of *E. coli* and S. aureus (Fig. 5d,k). Almost all the *E. coli* cells showed cytoplasmic injury to different degrees, regardless of whether the outer membrane was disintegrated or not. Nevertheless, no significant changes occurred in the cytoplasm of S. aureus cells until the cell wall fell off from the cells, which indicated the thicker cell wall of G+ bacteria protected the membrane permeability. In other words, the disruption of the cell wall is the primary mechanism of action on G+ bacteria. However, a double-edged-sword effect is the case here. Once losing the protection of the cell wall, the cell membrane of the G+ bacteria will be easily damaged and the cytoplasmic contents will leak out. In contrast, G− bacteria have three lines of defense in place for the cytoplasm. Therefore, the cytoplasmic contents of G− bacteria do not leak out easily.

Furthermore, CSS-Ag NPs tend to bind to the surface of G− bacteria (*E. coli*) rather than G+ bacteria (S. aureus), which may be an effect induced by the unique outer membrane of G− bacteria. As we know, the outer membrane is rich in polysaccharides, phosphatides, and protein^21^. The CSS-Ag NPs were coated with a polymer that contained abundant amino and hydroxyl groups. Therefore, the CSS-Ag NPs probably formed an interaction with the outer membrane. Some studies have found that the interaction between Ag NPs and the bacterial cell wall damages the permeability of the cell membrane^11, 19, 22^. This is confirmed by detachment of the outer membrane from the *E. coli* cell wall, which is a distinctive feature of outer membrane permeability change^23^. In addition, the low-density region in the cytoplasm of *E. coli* cells suggests the permeability of the cytoplasmic membrane can also be changed and silver ions penetrate into the cytoplasm to disturb the cellular function. Therefore, membrane permeability change is the primary mechanism of action for CSS-Ag NPs on G− bacteria.

### Morphological changes

**Figure 7:**
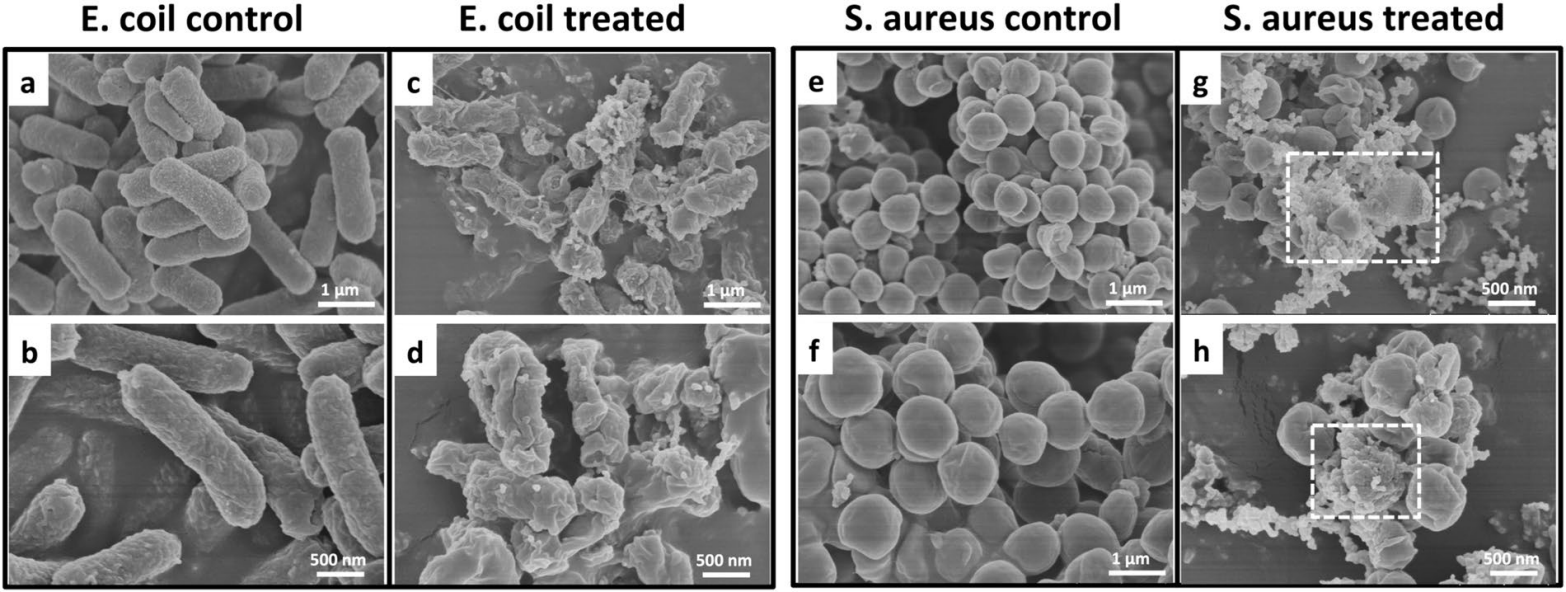
SEM images of bacteria treated with or without CSS-Ag NPs for 8 h. (**a,b**) Untreated E. coil; (**c,d**) treated E. coil; (**e,f**) untreated S. aureus; (**g,h**) treated S. aureus.

In order to confirm the results of TEM, the morphology of the bacteria was observed by SEM. The untreated bacterial cells (*E. coli* and S. aureus) remained intact and plump (Fig. 7a,b,e,f **)**. The cellular surface of *E. coli* after exposure to CSS-Ag NPs showed deep rill-like folds and distortion (Fig. 7c,d). The morphological changes may have been caused by the detachment of the membrane from the cell wall. The permeability of the cell membrane was damaged by CSS-Ag NPs, so the cells could not remain plump after dehydration via the ethanol series. Figure 7g,h shows the cytoplasmic contents of some treated S. aureus bacteria that was directly exposed without a cell wall/membrane, which corresponded with the TEM results.

### Membrane permeability changes

The results above revealed that CSS-Ag NPs killed G− bacteria (*E. coli*) by changing the membrane permeability, while the disruption in the cell wall was the primary mechanism of action on G+ bacteria. To verify these conclusions further, a *Bac*Light LIVE/DEAD membrane permeability kit was used to investigate the membrane permeability of the bacteria before and after exposure to CSS-Ag NPs. This assay used two DNA intercalating dyes: SYTO9 and propidium iodide (PI). Green fluorescent SYTO9 can stain viable cells by penetrating all of the membrane, whereas red fluorescent PI can only penetrate the permeabilized membrane and stains cells with a damaged cytoplasmic membrane^19, 24^. Therefore, the changes in membrane permeability can be easily evaluated under a fluorescence microscope.

**Figure 8:**
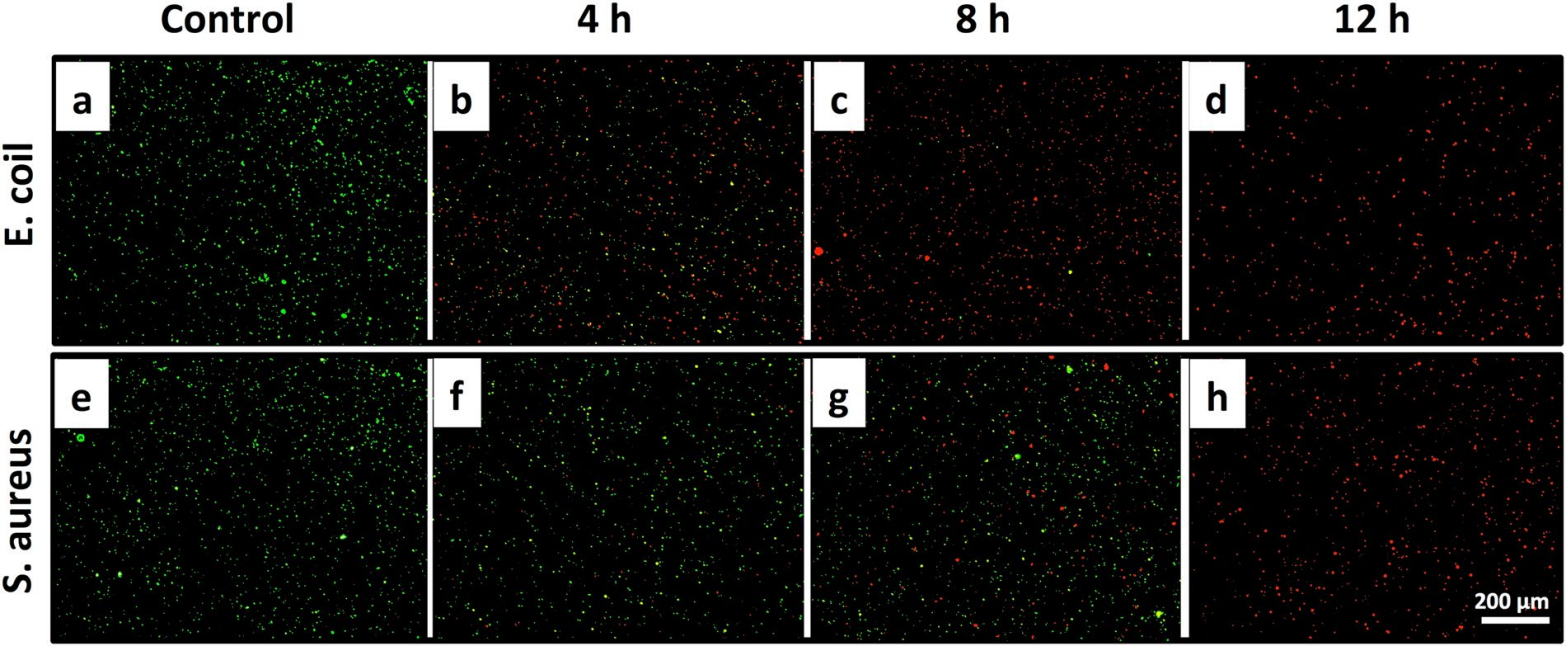
Fluorescence micrograph of *E. coli* and S. aureus treated with or without CSS-Ag NPs. After being treated with CSS-Ag NPs for 4 h (**b,f**), 8 h (**c,g**), and 12 h (**d,h**), respectively. The untreated *E. coli* and S. aureus served as a control group (**a,e**). The scale bar is 200 μm.

All of the untreated bacteria were stained with green fluorescence, which indicated that these cells maintained a normal membrane (Fig. 8a,e). Figure 8b–d shows *E. coli* cells after treatment with CSS-Ag NPs for 4, 8, and 12 h. A considerable number of the *E. coli* cells were stained by PI after just 4 h, and only a few of the cells were stained by SYTO9 after 8 h. All of the *E. coli* cells were stained red after 12 h exposure to CSS-Ag NPs. In contrast, Fig. 8f–h shows S. aureus cells after treatment with CSS-Ag NPs for 4, 8, and 12 h. Only a few of the cells were stained red after 4 h, and a considerable number of cells were still stained green after 8 h. A big change was observed at 12 h: almost all the cells were stained red.

The staining results revealed the membrane permeability changes of the bacteria after exposure to CSS-Ag NPs. Although the final images (12 h) for *E. coli* and S. aureus look the same, the changes in permeability over time are, in essence, different. For *E. coli*, an obvious change in the membrane permeability was observed after just 4 h, and the proportion of membrane-permeabilized cells increased slowly in the subsequent 8 hours. This indicated that the membrane permeability change is the primary mechanism of action for CSS-Ag NPs on G− bacteria, which is consistent with the TEM results.

On the other hand, S. aureus showed a tiny change in the membrane permeability in the first 4 h. A remarkable change was observed after 12 h, which may be because of a disruption in the cell wall. Based on the previous results, it is clear that the S. aureus cell wall protected the membrane permeability. It may take time for CSS-Ag NPs to destroy the thicker cell wall of S. aureus. Therefore, the membrane permeability of S. aureus was not changed by the nanoparticles in the first 4 h. When the cell wall was destroyed, the cell membrane was easily damaged and penetrated by PI. The above results verified the conclusion that the disruption of the cell wall is the primary mechanism of action on G+ bacteria.

### Leakage of cytoplasmic proteins

As shown in TEM and SEM images, when the S. aureus cell wall was destroyed, the intracellular (cytoplasmic) contents leaked out. However, there was no similar mechanism observed on the *E. coli* due to the three lines of defense for the cytoplasm. While electron microscopy provides useful insight into the leakage of contents, it still lacks quantitative and objective measurement. Some studies used the BCA protein assay kit to evaluate the leakage of proteins through the bacteria membrane^1, 11^.

**Figure 9:**
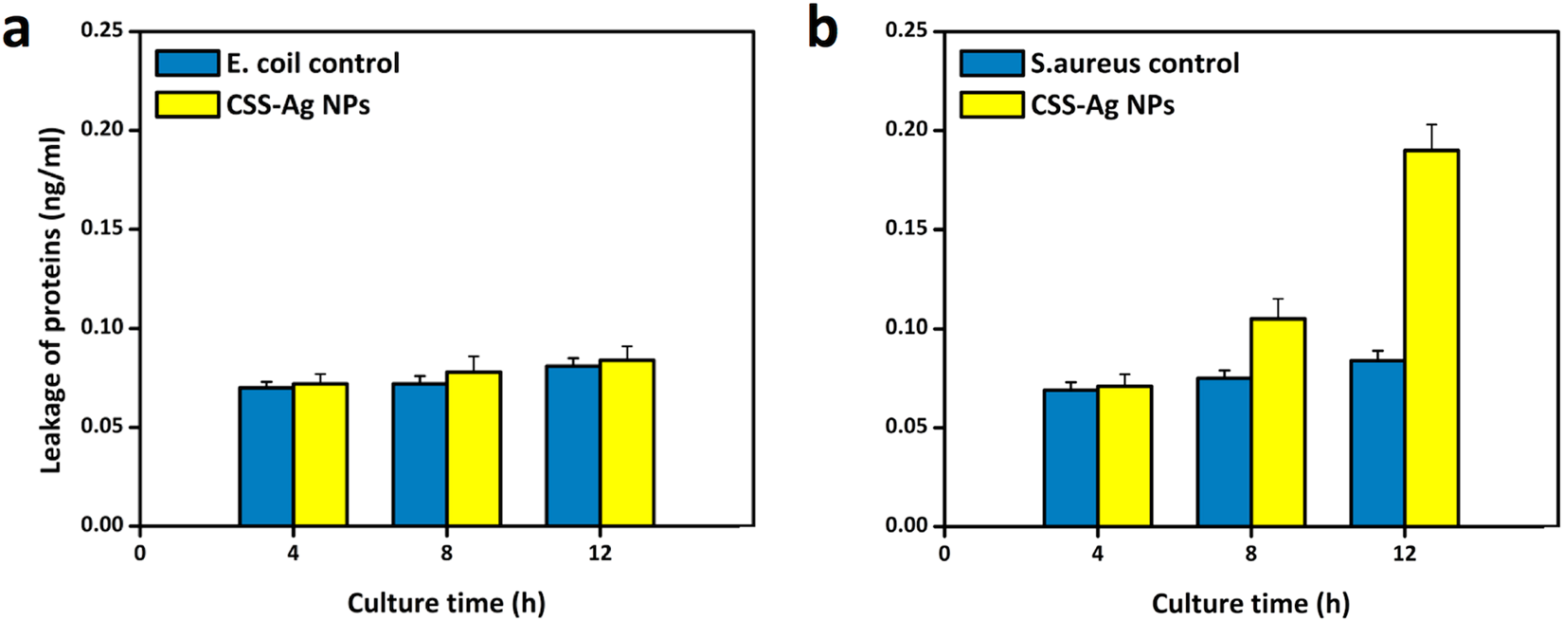
Leakage of cytoplasmic proteins after treated with CSS-Ag NPs using BCA assay. (**a**) S. aureus and (**b**) E. coil. The untreated *E. coli* and S. aureus served as a control group.

Figure 9 a shows the leakage of proteins in S. aureus treated with CSS-Ag NPs. As a control group, the untreated cells kept an almost constant amount of proteins over time, suggesting no leakage of cytoplasmic contents. The difference between treated cells and the control group was not significant at 4 h, suggesting the cell wall/membrane integrity. The difference became more obvious at 8 h and a huge gap was observed at 12 h. It is clear that a large amount of cytoplasmic proteins leaked out from S. aureus cells after 12 h exposure to CSS-Ag NPs. The result is consistent with the staining results.

In the control group, nearly no proteins were detected over time due to the leakage from the *E. coli* cells (Fig. 9b). The difference between the treated *E. coli* cells and the control group was slight at 4, 8 and 12 h, suggesting no leakage of proteins in the treated *E. coli*. Similar results were gained in TEM and SEM images.

## Conclusions

In this study, silver nanoparticles coated with catechol-conjugated chitosan were prepared using green methods. The resulting CSS-Ag NPs exhibited remarkable antibacterial activities at very low dosages, with an MBC of 14 μg/mL against *E. coli* and 25 μg/mL against S. aureus. It is clear that the CSS-Ag NPs exhibited a better antibacterial effect on G− bacteria rather than the G+ bacteria. In order to decipher the distinct antibacterial effects, we further investigated the changes in the cellular ultrastructure of the bacteria before and after exposure to CSS-Ag NPs. The TEM and SEM results revealed that the CSS-Ag NPs killed G+ and G− bacteria through different mechanisms. As G+ bacteria, S. aureus were killed by CSS-Ag NPs through disruption of the cell wall. The special thicker cell wall of G+ bacteria acts as a barrier protecting the cell from penetration of the silver nanoparticles and ions into the cytoplasm. Once losing cell wall integrity, the cell membrane of G+ bacteria is easily damaged and the cytoplasmic contents leak out, which has been confirmed by the leakage of proteins in assay. On the other hand, the CSS-Ag NPs adsorbed on the surface of *E. coli* cells and formed an interaction with the outer membrane, which damaged the membrane permeability. The change in permeability allowed the silver ions to penetrate into the cytoplasm and disturb the cellular function. The Live/Dead staining assay confirmed that the membrane permeability change is the primary mechanism of action for CSS-Ag NPs on G− bacteria. In conclusion, CSS-Ag NPs exhibited distinct, species-specific mechanisms due to the structural differences in cell wall of G+ and G− bacteria. G− bacteria are more easily killed by CSS-Ag NPs due to their lack of a thicker cell wall and the consequent adsorption of nanoparticles in the special outer membrane. Our study offers an in-depth understanding of the antibacterial behaviors of CSS-Ag NPs and ultimately provides insights into optimizing the design of Ag NPs for treatment of bacterial infections.

## Methods

### Materials

Chitosan (M_η_ 30 kDa, 80% degree of deacetylation) was purchased from Zhejiang Gold Shell Pharmaceutical Co. Ltd. 3,4-dihydroxy hydrocinnamic acid and 1-ethyl-3-(3-dimethylaminopropyl)-carbodiimide hydrochloride (EDC) were purchased from Sigma-Aldrich. Silver nitrate was purchased from Acros Organics. Bicinchoninic acid (BCA) protein assay kit and Live/Dead bacterial viability kit were purchased from Beyotime Institute of Biotechnology. All the other reagents were analytical grade.

### Synthesis and characterization of CSS and CSS-Ag NPs

According to our previous reported work, catechol-conjugated chitosan was synthesized in a standard EDC procedure and the CSS-Ag NPs were prepared using green methods^9^. The CSS chemical structure was analyzed by ^1^H NMR (Bruker Avance, 600 MHz, D_2_O) and FT-IR (Nicolet Avatar 230 spectrometer). The phase structure of CSS was analyzed by X-ray diffraction (XRD, Rigaku D/Max-2550 PC, Japan). The silver concentration of the CSS-Ag NPs was measured by atomic absorption spectrophotometer (AAS, Hitachi 180-50, Japan). The distribution of the nanoparticles was determined in the UV-vis spectrum (Hitachi U-1000, Japan), TEM, and Malvern Zetasizer Nano ZS (United Kingdom).

### Bacterial culture

The model microorganisms used were *E. coli* and S. aureus. The bacterial strains were cultured in nutrient broth and incubated in a shaking incubator at 37 °C overnight. The bacterial cells were brought into log phase by reinoculating the overnight culture 1:100 into fresh media and growing at 37 °C in the shaking incubator for several hours until an optical density of 600 nm (OD 600) of 0.1 was reached^12^.

### Minimum bactericidal concentration (MBC)

The MBC was defined as the minimum concentration sufficient to kill bacteria *in vitro*, which is used to quantitatively evaluate the antibacterial activities^25^. In this method, 2 mL CSS-Ag NP solutions containing different concentrations of silver were prepared in sterile test tubes by a serial dilution method. Then, bacterial cells were collected by centrifugation (5000 rpm, 5 min) and diluted with sterile phosphate saline buffer solution (PBS, pH 7). Two mL bacterial suspensions (OD 600 = 0.1) were added into the test tubes and incubated for 12 h at 37 °C. The number of viable bacterial cells in the above precipitate was analyzed by counting colony-forming units (CFUs). Briefly, 50 μl gradient dilutions of each culture were plated in triplicate LB-agar plates, incubated at 37 °C for 24 h, and the formed colonies were counted and recorded. The lowest concentration at which colony formation remained absent was taken as the MBC.

### Preparation of bacteria treated with CSS-Ag NPs

The bacterial suspension (OD 600 = 0.1) was cocultured with CSS-Ag NPs (at the minimum bactericidal concentration) for 4, 8, and 12 h at 37 °C, respectively. The untreated bacterial suspension served as a control group. The coculture supernatant was then harvested by centrifugation (5000 rpm, 5 min). The precipitate was collected for subsequent measurements.

### Ultrastructure of the bacteria

For TEM observation of the bacteria, the precipitates (cocultured with or without CSS-Ag NPs for 8 h) were fixed with 2.5% glutaraldehyde solution overnight at 4 °C. The cells were then washed in phosphate buffer (PB) and resuspended in 1% agarose. The agarose-embedded cell pellets were fixed in 2% osmium tetroxide for 2 h and washed three times in PB. The cells were then dehydrated via an ethanol series (50%, 70%, 80%, 90%, and 100%, respectively) and embedded in Epon/Araldite resin (polymerization at 60 °C for 48 h). Ultrathin sections containing cells were placed on the grids, stained with uranyl acetate, and lead citrate solution, and examined under the TEM (Hitachi H-7500)^26^.

### Morphology of the bacteria

For SEM observation, the precipitates (cocultured with or without CSS-Ag NPs for 8 h) were fixed with 2.5% glutaraldehyde solution overnight at 4 °C. The cells were then dehydrated with 50%, 70%, 80%, 90%, and 100% ethanol for 20 min each. The samples were dried, coated with gold, and then examined with a SEM (JSM 6310, Jeol Ltd., Akishima, Tokyo, Japan)^27^.

### Live/Dead bacterial staining assay

A *Bac*Light LIVE/DEAD membrane permeability kit was used to determine the membrane permeability of the bacteria. A solution containing a mixture of SYTO^®^9 and propidium iodide dyes was prepared according to the manufacturer’s instructions. The bacterial precipitate was prepared as described above and mixed with the fluorescent dyes for 15 min. Then the bacterial suspension was centrifuged (5000 rpm, 5 min) and washed three times with PBS. The samples were dropped on the microscope slides (5 μL for each sample), covered with a cover slip, and imaged by a fluorescence microscope (LSM 510, Carl Zeiss). Images were obtained by a 10× objective lens^28^.

### BCA protein assay kit

The leakage of proteins through the bacteria membrane was detected according to previous methods^11^. The coculture supernatant was prepared as described above and the concentration of proteins was determined by BCA protein assay kit. Each experiment was performed in triplicate.
